# News and Notes

**Published:** 1905-09

**Authors:** 


### *NEWS AND NOTES.*

---

DR. C. F. BENSON has been quite ill.

---

DR. AND MRS. JOHN HURT announce the birth of a son.

---

DR. AND MRS. ELKIN returned to the city the 1st of September.

---

DR. L. C. ROUGHLIN and Miss Lena Weinberg were married August 22d.

---

DR. and MRS. J. H. CRAWFORD have been in London, Vienna and Paris.

---

DR. WILLAM PURKS died at his home in Greensboro, Ga., August 20th.

---

DR. W. A. CROWE has been visiting in Chicago and other points in the Northwest.

---

DR. AND MRS. W. S. KENDRICK have returned to city from Boston and New York.

---

DR. CLAUD SMITH has returned from an extended trip in the Yellowstone Park, Oregon and Alaska.

---

DR. AND MRS. C. E. MURPHEY and little daughter, Mary, were at Borden-Wheeler Springs for a short stay.

DR. and MRS. FREDERICK G. HODGESON have announced the birth of a daughter, Margaret Crocker. They will remain at their summer home at Mt. Airy until October 1st.

---

MR. D. SIMON, a barber of Baltimore, has advertised that he will give $300 for a complete cure of his cousin, who has diabetes. There were eleven sanguine aspirants for the $300.

---

DR. J. N. LECONTE has sailed for Europe to spend several months in the study of stomach and intestinal diseases. While abroad he will furnish this journal with some interesting letters on general medical matters.

---

A PLAGUE of mosquitoes is said to be widespread in London. Formerly they were limited largely to the region of the docks but this summer they are being found all over the city in enormous numbers.

---

THE epidemic of typhoid fever in Washington, D. C., has assumed serious proportions, more than 300 cases having been reported since July 1st. The new filtration plant is now in operation and it is to be hoped that the conditions may soon be thoroughly under control.

---

DR. D. T. D. BERRY, of the United States Marine Hospital Corps, at work in New Orleans, has been taken down with yellow fever. He was supposed to be an immune and had charge of the inspection service in the Italian district. The latest dispatches report a rapid improvement in his condition.

THE semi-annual report of Birmingham's health officer shows the death-rate among the negroes of the city to be much larger than the birth-rate. The negro birth rate for 1,000 is 21.12 and the death rate 33.32. The crowded quarters in which they live, which makes tuberculosis very prevalent, is assigned as the cause.

---

AN outbreak of cholera has been reported in Manila. It is supposed to have been brought in green vegetables from Hong-Kong. Several soldiers have died at Camp McKinley, which is now quarantined. The Sixteenth Regiment has recently arrived there from Fort McPherson. Heroic measures are being taken to place it under control.

---

MANY prominent physicians and surgeons from Great Britain, the United States and Canada were present at the annual meeting of the Canadian Medical Association, which met in Halifax, N. S. Among those making addresses were, Francis Craig, of Edinburgh ; Dr. Howard Kelly, of Baltimore ; Dr. E. N. Cushing, of Boston, and Dr. George Armstrong, of Montreal.

---

AT the next meeting of the Mississippi Valley Medical Association to be held at Indianapolis, Ind., October 10, 11, 12, the annual addresses will be delivered by Dr. Arthur R. Edwards, of Chicago, and Dr. W. D. Haggard, of Nashville, Tenn.

Dr. Edwards has chosen for the subject of his address, "Certain Phases of Uremia, Their Diagnosis and Treatment," and Dr. Haggard will discuss in his address, "The Present Status of Surgery of the Stomach." In addition to these addresses there will be the annual address of the President, Dr. Branford Lewis, of St. Louis.

A cordial invitation is extended to every physician in the valley to attend this meeting for which a large number of interesting and valuable papers has been promised.

TRI-STATE (ALA., GA. & TENN.) MEETING.—The seventeenth annual meeting of the Tri-State Medical Society of Alabama, Georgia and Tennessee will be held in Chattanooga, Tuesday, Wednesday and Thursday, September 26, 27 and 28, 1905.

A rate of one fare for the round trip has been secured on account of fall meeting of the Chattanooga Fair Association. This organization will have a Horse Show and other attractions, September 26th to 30th.

Membership to this Association is open to all members of the profession in good standing, and a most cordial invitation is extended to all such medical men.

A program of unusual merit is already assured—and those who have not yet sent the subject of their papers should do so at once to the Secretary, Dr. Raymond Wallace, Chattanooga, Tennessee.

---

DR. T. D. CROTHERS, of Hartford, Conn., Superintendent of Walnut Lodge Hospital, has accepted an invitation to deliver the first oration in the Norman Keer Memorial Lectureship at London, Eng., October 10, 1905. Dr. Keer will be remembered as an eminent London physician who made a special study of inebriety, alcoholism and other drug disorders. He wrote several excellent books on this subject, and was instrumental in securing the enactment of laws for the control of inebriates, and the promotion of hospitals for their care throughout Great Britain. He founded the British Society for the study of inebriety in 1884, and this Society and his friends have organized a Memorial Lectureship for the yearly orations on his life and work. It is a very pleasant recognition of the progress of medical science in this country that an American physician should be invited to deliver the first lecture.

